# Design of high-performance entangling logic in silicon quantum dot systems with Bayesian optimization

**DOI:** 10.1038/s41598-024-60478-9

**Published:** 2024-05-02

**Authors:** Ji-Hoon Kang, Taehyun Yoon, Chanhui Lee, Sungbin Lim, Hoon Ryu

**Affiliations:** 1 Division of National Supercomputing, Korea Institute of Science and Technology Information, Daejeon, 34141 Republic of Korea; 2 Artificial Intelligence Graduate School, Ulsan National Institute of Science and Technology, Ulsan, 44919 Republic of Korea; 3 Department of Artificial Intelligence, Korea University, Seoul, 02841 Republic of Korea; 4 Department of Statistics, Korea University, Seoul, 02841 Republic of Korea

**Keywords:** Quantum information, Quantum simulation, Qubits, Quantum information, Computational science

## Abstract

Device engineering based on computer-aided simulations is essential to make silicon (Si) quantum bits (qubits) be competitive to commercial platforms based on superconductors and trapped ions. Combining device simulations with the Bayesian optimization (BO), here we propose a systematic design approach that is quite useful to procure fast and precise entangling operations of qubits encoded to electron spins in electrode-driven Si quantum dot (QD) systems. For a target problem of the controlled-X (CNOT) logic operation, we employ BO with the Gaussian process regression to evolve design factors of a Si double QD system to the ones that are optimal in terms of speed and fidelity of a CNOT logic driven by a single microwave pulse. The design framework not only clearly contributes to cost-efficient securing of solutions that enhance performance of the target quantum operation, but can be extended to implement more complicated logics with Si QD structures in experimentally unprecedented ways.

## Introduction

Modern information and communication technology (ICT) owes a great deal to silicon (Si) material on which electronic devices are integrated, enabling the enormous increase in computing power and storage capacity. The territory of Si material is being extended to emerging quantum information technology due to the matured industrial-standard fabrication process; for example, the quantum logic devices have been integrated into a single Si wafer^1–5^. Electrically defined Si quantum dot (QD) system has already proved its manufacturability and feasibility as a versatile platform for implementation of quantum logic gates^1,6–15^. Since Loss & DiVincenzo proposed the state-of-art concept for implementation of universal gates using QD-confined spins^16^, researchers have reported stable addressing of individual quantum bit (qubit)^10,14,15^, implementation of a SWAP & a controlled-Z (CZ) gate^11,14,15^, and a fast CNOT logic driven with a single microwave pulse^12^. In spite of remarkable progresses achieved by preceding works, the Si-based qubit technology is still generally behind the ones based on superconductors^17,18^ or trapped ions^19,20^. Elaborated efforts are still required to resolve design issues such as fidelity-degradation driven by material-inherent noises^21–25^, instability of spin states stemming from the Rashba effect in nanoscale layers^26,27^, and scalable implementation of entangling logics^14,15^.

Computer-aided simulations are essential to resolve the above-mentioned design problems since they can not only handle enormous design variations that are practically impossible to be uncovered with experiments, but also improve existing designs in experimentally unprecedented ways^25,28–32^. However, finding design solutions often involves huge amount of trial-and-errors and a massive set of simulations needs to be conducted with parameter sweeps until results satisfy design criteria. In our previous studies^25,28^, for example, a charge stability diagram is simulated with 2,500 sets of electrical biases to find the condition for qubit initialization in a Si double QD (DQD) system, spending several thousand core-hours. Moreover, even though solutions are obtained, their quality may deteriorate if search cases are limited due to the design complexity. Naive random search hardly considers complicated nonlinear relations among design parameters in physical systems; hence, it becomes difficult to get optimal designs without an appropriate case-sampling approach.

So in this work, we devise a design strategy based on the Bayesian optimization (BO)^33^, a principled methodology which efficiently optimizes a black-box function by making decisions between exploration and exploitation in a data-driven way when the function is costly. To bypass time-consuming case-by-case device simulations, BO estimates the possibility of finding design solutions in an exploratory domain with the surrogate function that is augmented with a restricted number of simulated data. To find the optimal solution more efficiently, BO samples data points with the acquisition function that considers the trade-off between exploration and exploitation, differently from the brute-force approach where sampling points are searched manually^34^. As a target problem of the proposed design approach, we use the experimentally reported Si DQD structure^12^ where a CNOT operation is implemented with a single-step control of time-varying magnetic pulses. Employing our in-house device simulator^28^ as a black-box function of BO, we focus on finding spin resonance frequencies and inter-spin exchange interaction that minimize the operating time while maintaining the fidelity larger than the given criterion. With device simulations, we find the physical design in Si DQD structures that reproduces the BO-driven results, verifying the practicality of our approach that in principle contributes to saving the working load required to find design solutions compared to the case when only device simulations are employed.

## Methods

### Computational flow of the proposed design approach

**Figure 1:**
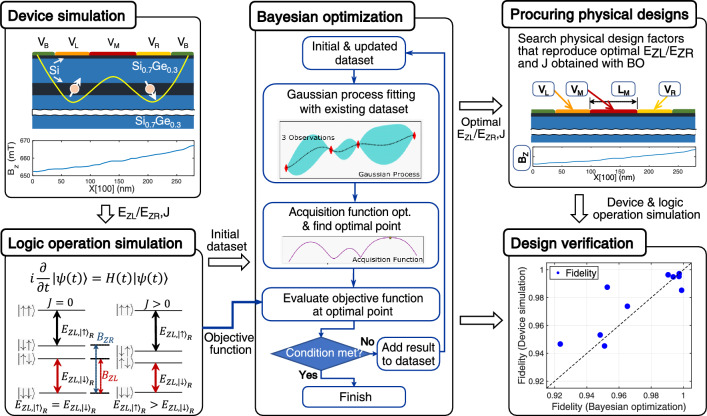
Overall process of the design framework. Quantum logic operations of the silicon (Si) double quantum dot (DQD) platform are modeled with device simulation (left upper) and logic operation simulation (left lower). From device simulations of DQD structures, we obtain Zeeman-splitting energy ($E_{ZL}$ and $E_{ZR}$) and exchange interaction energy (*J*) that are used to constructs the Heisenberg Hamiltonian of electron spins. Time responses of spin qubits can be obtained by solving time-dependent Schrödinger equation described with the Heisenberg Hamiltonian. The set of ($E_{ZL}$, $E_{ZR}$, *J*), which can implement the fastest CNOT operation under user-defined criteria of operational fidelity, is determined by the Bayesian optimization (BO) technique that takes the logic operation simulation block as a black-box objective function. To verify the feasibility of BO-driven solutions, we find physical designs of DQD structures (right upper), and examine how results obtained by device simulations and BO are correlated (right lower).

Figure 1 illustrates the entire design process that is devised to study a single-step CNOT operation with BO. The process starts with the *device* *simulation* block where potential energy profile and electron spin states of the Si DQD structure are self-consistently determined with our in-house device simulation tool based on a hybrid utilization of the bulk physics and electronic structure calculations. Once energetic positions and charge distributions of the electron spin states are determined, we calculate Zeeman-splitting energies of both QDs ($E_{ZR}$, $E_{ZL}$) and exchange interaction (*J*) that are the final outputs of the first block. With $E_{ZR}$, $E_{ZL}$ and *J*, the next block (*logic* *operation* *simulation*) constructs the Heisenberg Hamiltonian to simulate the 2-qubit time responses and the outputs of this block become the operation time ($t_{CNOT}$) and corresponding fidelity (*F*) of a single-step CNOT operation. We note that in-depth description on computational details for the above-mentioned two blocks is available in our latest works^25,28^.

Taking the logic operation simulation block as a black-box objective function, BO is conducted to minimize $t_{CNOT}$ under user-defined fidelity criteria against the target design variables ($E_{ZR}$, $E_{ZL}$ and *J*) given as inputs of the objective function. For initial observation data that is required to begin the BO process, we find the realistic conditions of inputs ($E_{ZR}$, $E_{ZL}$ and *J*) and outputs ($t_{CNOT}$ and *F*) that faithfully reproduce the experimental results^12,28^. Once we get the solutions, *i*.*e*., $E_{ZR}$, $E_{ZL}$ and *J* that minimize $t_{CNOT}$ whilst maintaining *F* larger than given values, we find physical designs of a Si DQD system to verify the feasibility of optimal design factors searched by BO, because BO itself does not know whether they indeed can be implemented in a real Si DQD system.

### Bayesian optimization

BO is an iterative algorithm that optimizes a black-box objective function whose computation is costly. To mitigate the cost problem of computation, BO handles two tasks to negotiate the accuracy of prediction with the number of function calls. During iterations, BO adopts (a) a surrogate function to approximate the objective function with a lower computational cost than the actual objective function, and (b) an acquisition function to quantify the uncertainty of each prediction for efficient optimization, thereby calling the objective function only for the most probable candidate point. In the optimization loop, BO evaluates the surrogate function at randomly selected candidate points and chooses the best candidate as a sampling point using the acquisition function, where the exact value at the sampling point is then calculated with the objective function. The optimization loop iterates until the solution satisfies the design criteria. For the surrogate function, we adopt Gaussian Process (GP) regression^35^:

1 $$\begin{aligned} g(\textbf{x}) \sim \mathscr{G}\mathscr{P} :=\mathscr {N}(m(\textbf{x}), k(\textbf{x}, \textbf{x}')), \end{aligned}$$

where *m* and *k* represent GP mean and covariance function, respectively, while $\textbf{x}$ and $\textbf{x}'$ denote a data point. Without loss of generality, we can assume $m(\textbf{x}) = 0$ for GPs.

The major advantage of employing GP as the surrogate function is that there exist analytic formulae for mean and covariance of the posterior distribution, which refer to the probability distribution of unobserved variables based on a collection of observed data. For function values $\textbf{y}_T = [y_1, \dots , y_T]^T$ at observed points $A_T = \{\textbf{x}_1, \dots , \textbf{x}_T\}$, where $y_t = f(\textbf{x}_t)$, the posterior over *f* is also Gaussian. The posterior mean $\mu _T(\textbf{x})$, covariance $\Sigma _T(\textbf{x}, \textbf{x}')$ and variance $\sigma _T^2(\textbf{x})$ can be obtained as follows^35^:

2 $$\begin{aligned} \mu _T(\textbf{x})&= \textbf{k}_T(\textbf{x})^T (\textbf{K}_T)^{-1} \textbf{y}_T, \end{aligned}$$

3 $$\begin{aligned} \Sigma _T(\textbf{x}, \textbf{x}')&= k(\textbf{x}, \textbf{x}') - \textbf{k}_T(\textbf{x})^T (\textbf{K}_T)^{-1}\textbf{k}_T (\textbf{x}'), \end{aligned}$$

4 $$\begin{aligned} \sigma ^2_T(\textbf{x})&= \Sigma _T(\textbf{x}, \textbf{x}), \end{aligned}$$

where $\textbf{k}_T(\textbf{x}) = [k(\textbf{x}_1, \textbf{x}), \dots , k(\textbf{x}_T, \textbf{x})]^T$ and $\textbf{K}_T =[k(\textbf{x}_i, \textbf{x}_j)]_{\textbf{x}_i, \textbf{x}_j \in A_T}$ is the positive definite kernel matrix.

Given variance and expectation of the posterior distribution, the acquisition function suggests an optimization strategy to choose the next sampling point, considering the trade-off between exploration and exploitation. There are several candidates for the acquisition function, such as probability of improvement (PI)^36^, expected improvement (EI)^33^ or Gaussian process upper confidence bound (GP-UCB)^37^. Since Srinivas *et* *al*. reported that GP-UCB reduces the average number of function calls to find global optimum of black-box functions by quantifying the uncertainty of the parameter search procedure^37^, here we adopt GP-UCB as the acquisition function that is formulated as follows:

5 $$\begin{aligned} {\textbf {x}}_{i+1} = \arg \max \limits _{{\textbf {x}} \in {\mathscr {X}}} \text {UCB}({\textbf {x}}; \lambda ) = \arg \max \limits _{{\textbf {x}} \in {\mathscr {X}}} \mu _{i}({\textbf {x}}) + \lambda \Sigma _{i}({\textbf {x}}), \end{aligned}$$

where $\mu$ and $\Sigma$ are GP posterior mean and covariance function that are shown in Eqs. (2) and (3), respectively. $\lambda$ is a constant for balancing between exploration and exploitation. ${\mathscr {X}}$ denotes the parameter space. The sampling point ${\textbf {x}}_{i+1}$ is determined for the ($i+1$)th iteration step with Eq. (5). In general, BO employing GP incurs a time complexity of $O(T^3)$ for updating the GP model, attributed to the covariance matrix inversion, where *T* denotes the number of observations. The computational endeavor of determining the next point for sampling, primarily through the optimization of the acquisition function, is typically less burdensome than the model update cost. Notably, the time complexity of GP-based methods like GP-UCB significantly undercuts that of the grid search strategy - $O(k^n)$, with *n* denoting the number of parameters and *k* the number of potential parameter values^38^. Designed to curtail the cost of function evaluations required to closely approximate the global optimum, BO showcases superior efficiency over grid search, particularly in scenarios where objective function assessments are exhaustively demanding^37^.

**Algorithm 1:**
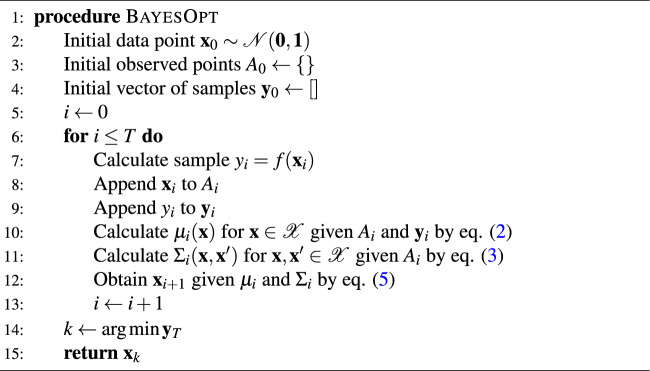
Process of Bayesian optimization.

**Figure 2:**
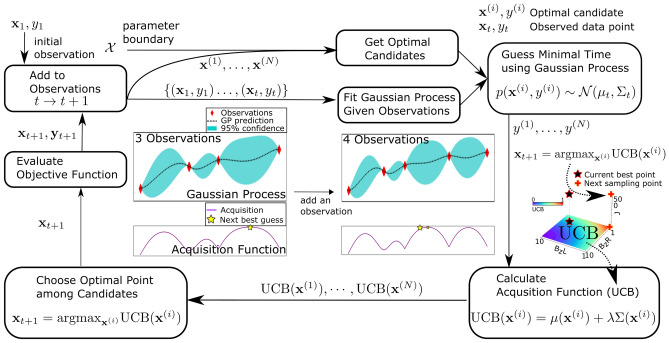
Schematic flow chart of the Bayesian optimization (BO) process. The input variables are design factors of CNOT logic ($\textbf{x}$), *i*.*e*., Zeeman-splitting energies ($E_{ZL}$, $E_{ZR}$) and exchange interaction (*J*), where the target output to be optimized (*y*) is the CNOT operation time ($t_{CNOT}$). In each iteration of BO, the surrogate function is fitted to the observations of $\textbf{x}$ and *y* that are accumulated so far, and the minimal *y* is predicted by evaluating the updated surrogate function at randomly chosen design factor candidates ($\textbf{x}^{(i)}$). The acquisition function based on the upper confidence bound (UCB) is then employed to decide the design factor candidate ($\textbf{x}_{t+1}$) that will be used as an input of the objective function. Corresponding output ($y_{t+1}$), with $\textbf{x}_{t+1}$, is added as a new observation. As iteration process continues, uncertainty within the search space is reduced due to the increased number of observations, so the updated *y* and $\textbf{x}$ can become more desirable.

The whole BO process is schematically shown in Fig. 2. Here, the target objective function is a mapping $f: \textbf{x} \in \mathbb {R}^3 \rightarrow y \in \mathbb {R}$, where ${\textbf {x}}$ is a set of values of the design factors and $y = f(\textbf{x})$ indicates the fastest $t_{CNOT}$ that can be secured with the fidelity threshold. Initially, we select ${\textbf {x}} \sim {\mathscr {N}}(\textbf{0}, \textbf{1})$ from the parameter space ${\mathscr {X}}$ and sample the corresponding objective function value *y*. The GP posterior distribution is then calculated with Eqs. (2) and (3) including the newly sampled data point. From the obtained posterior distribution, the acquisition function based on GP-UCB is evaluated over the random samples of the entire search domain and the next sampling point is determined where the acquisition function is maximized as indicated in Eq. (5). The BO process discussed so far is summarized in Algorithm 1. In numerical experiments, we define ${\textbf {x}} = [E_{ZL}, E_{ZR}, J]^T\in \mathscr {X}$ where ${\mathscr {X}} = [15, 24] \times [15, 24] \times [0.3, 100]$, *i*.*e*., the range of $E_{ZL}, E_{ZR}, J$ are set to [15, 24]GHz, [15, 24]GHz and [0.3, 100]MHz, respectively. In the line 6 and 10 of Algorithm 1, we set the maximum number of iteration steps (*T*) and $\lambda$ for the acquisition function to 50 and 10, respectively.

## Results and discussion

**Figure 3:**
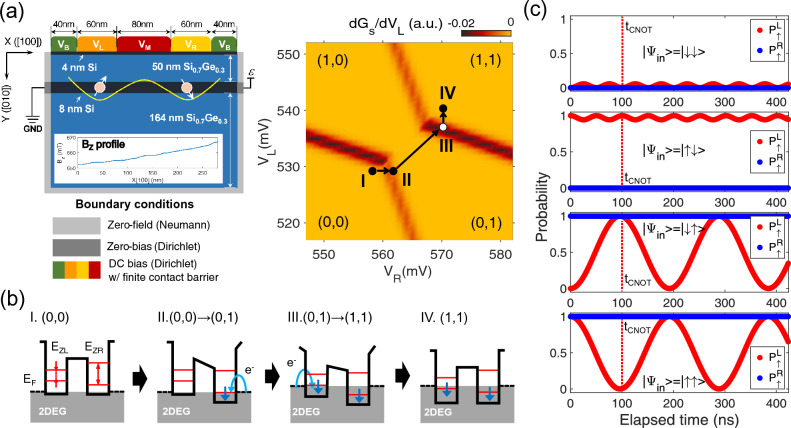
System initialization and CNOT operation. (**a**) Target double quantum dot (DQD) structure and charge stability diagram showing electron population in each QD. QDs are formed in the middle silicon (Si) layer with the vertical confinement created by band offset between Si & silicon-germanium (SiGe) layer and the lateral confinement created by controlling biases on top electrodes (two barrier gate biases ($V_B$) and a left/middle/right gate bias ($V_L/V_M/V_R$)). A lateral distribution of the static magnetic field ($B_Z$) that is generated from the external magnet^40^, is shown in the inset figure. A charge stability diagram is simulated with the model structure as a function of $V_L$ and $V_R$ with $V_M$ = 400 mV and $V_B$ = 200 mV. The path from point I to point IV indicates the initialization sequence of the DQD system. The simulated charge control is in excellent agreement with the experimentally reported result^12,25^. (**b**) A conceptual illustration that shows the initialization sequence. By controlling $V_L$ and $V_R$ biases from the empty QDs (point I), the right QD (point II) and left QD (point III) are sequentially occupied with a single electron. The initialization is completed at point IV where both QDs are filled. (**c**) Two-qubit time responses obtained with four input states at $V_L$ =540 mV, $V_R$ = 570 mV and $V_M$ = 408 mV. The first CNOT operation is implemented at *t* = 100.4 nsec ($t_{CNOT}$) where the spin in the right QD is used as a control qubit.

A prerequisite for BO is to secure initial observation data which here consist of design parameters ($E_{ZL}, E_{ZR}, J$) that reproduce the experimentally reported single-step CNOT operation^12^. The first step for finding them is to figure out the bias points which initialize qubit states for subsequent gate operations in the target DQD structure where qubits are encoded to the electron spin in the ground state of each QD, *i*.*e*,  $|0\rangle$ for the down-spin ($|\downarrow \rangle$) and $|1\rangle$ for the up-spin ($|\uparrow \rangle$) state. For this purpose, a full charge stability diagram is simulated to explore the ranges of biases where the down-spin state of each QD is filled with one electron, and Fig. 3a shows the results with an illustration of the target Si DQD structure (the middle gate bias ($V_M$) and barrier gate bias ($V_B$) are set to 400 mV and 200 mV, respectively). Being plotted as a function of the left ($V_L$) and right ($V_R$) gate biases, the charge state here is identified with two numbers that indicate the electron population in the left and right QD. To help readers understand how the DQD system works, a control path to initialize the DQD structure to the (1,1) state from the empty state is marked up with black arrows from point I to point IV in Fig. 3a. Increasing $V_R$ from point **I** lowers the energy level of the ground state in the right QD, and eventually fills the right QD with an electron at point **II** where the down-spin ground state in the right QD touches the Fermi energy. By increasing both $V_L$ and $V_R$ from point **II** to point **III**, we can also fill the down-spin ground state of the left QD. At the point **IV**, the system is biased symmetrically, which is known to be good for noise-robustness of upcoming qubit operations^22^. The above-mentioned process of charge transfer is conceptually illustrated in Fig. 3b, where the energy level of QDs from point **I** to **IV** are drawn in connection with the Fermi energy ($E_F$) of the source & drain two-dimensional electron gases. Once the DQD system is initialized to a $|\downarrow \downarrow \rangle$ state with $V_L$ = 540 mV and $V_R$ = 570 mV (point **IV**), the single-step CNOT operation is simulated at $V_M$ = 408 mV where *J* is large enough to drive entangling operations^25^. ($E_{ZL}$, $E_{ZR}$, *J*) obtained with device simulations at this point reads (18.287GHz, 18.501GHz, 19.3MHz). Figure 3c shows the simulated two-qubit responses, where the fastest CNOT operation is accomplished at $t_{CNOT}$ = 100.4 nsec with *F* = 98.34 %. The values of $t_{CNOT}$ and *F* are employed as the initial observation data for BO as well as with the values of $E_{ZL}$, $E_{ZR}$ and *J*.

**Figure 4:**
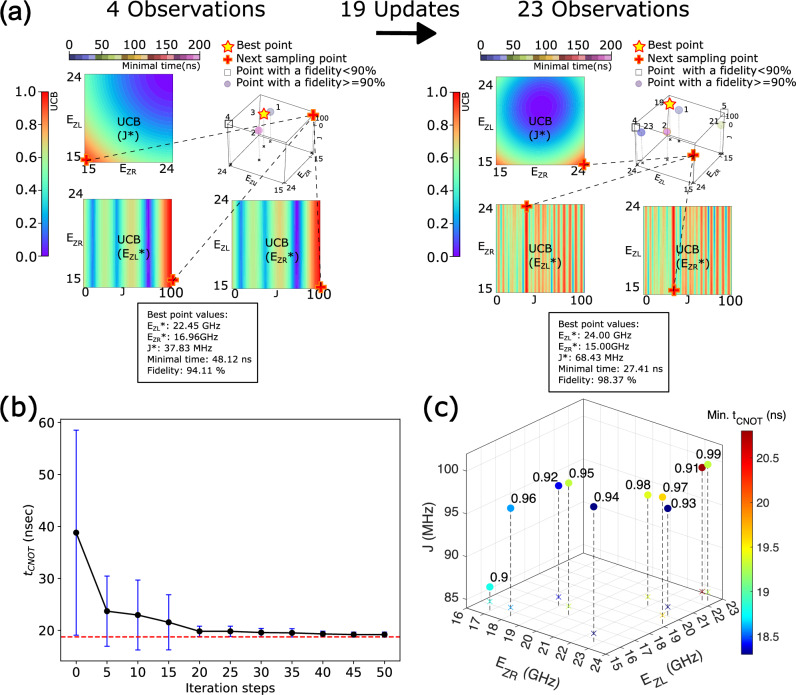
Evolving the Bayesian optimization (BO) process to search design factors and corresponding results. (**a**) Snapshots of BO process with 4 observations (left) and 23 observations (right). Scattered points in the 3D plot represent sampled points at each iteration step, while some of the sampled points are omitted for visibility in the lower subfigure. Each sampled point is differently colored according to the minimal operation time of CNOT logic that satisfies the fidelity criteria. Colors of contour plots indicate the Upper Confidence Bound (UCB) at the current step, showing how UCB values change depending on correlation between control variables. The best point in terms of the minimal operation time within the observations and the next sampling point are marked with yellow-star and red-cross symbols, respectively. (**b**) Convergence pattern of the minimal operation time ($t_{CNOT}$). Error bars show the standard deviation of 10 $t_{CNOT}$ values that are obtained with different random seeds. The red dotted line shows the average $t_{CNOT}$ ($\simeq$ 18.8nsec) that is obtained with 200 iterations, to which the solution converges within 50 iterations for all the 10 cases. (**c**) Zeeman-splitting energies ($E_{ZR}$, $E_{ZL}$) and exchange interaction (*J*) that are driven by BO under the fidelity threshold ranging from 90% to 99%. Being colored according to the $t_{CNOT}$ value, dot symbols represent design factors that drive the fastest operation within the threshold.

Figure 4a presents two snapshots describing the evolving process of BO, which starts with the initial observation data of the target design factors. Colors of the three 2D contour plots show the UCB value as a function of $(E_{ZL}, E_{ZR})$, $(E_{ZL}, J)$, and $(E_{ZR}, J)$, respectively. The uncertainty of the GP prediction with respect to the exact result of the objective function is high in the red area where the next sampling point are decided. The phenomenon that UCB values over *J* fluctuate much more strongly than those over $E_{ZL}$ and $E_{ZR}$ do, implies that *J* is the most sensitive parameter among the target design factors affecting $t_{CNOT}$ and *F*, being well connected to the fact that the fidelity of a single-step CNOT operation is highly sensitive to *J*^39^. Figure 4b shows how $t_{CNOT}$ converges as the BO process evolves. Here, a total of 10 different random seeds are tested to examine how the random seed affects the convergence speed, and the converged value of the average $t_{CNOT}$ after 200 iterations is marked at 18.8 nsec as a dotted red line. What we find is that $t_{CNOT}$ converges to the baseline before 50 iterations in all the cases so the maximum iteration number (*T* in Algorithm 1) we set is large enough to conduct experiments. Figure 4c shows the design factors obtained with BO with different thresholds in fidelity ranging from 90% to 99% with a step of 1%, where the resulting $t_{CNOT}$’s are indicated with colored dot symbols (see the Supplementary Information document for results obtained with a threshold of 99.9% and 99.99%). Here, the optimal design factors turn out to be almost evenly distributed across the search space of $E_{ZL}$ and $E_{ZR}$, while they are concentrated in the range of [85, 100]MHz in the case of *J*. Results here indicate that the gating speed of a single-step CNOT operation is correlated to *J* more strongly than to $E_{ZL}$ and $E_{ZR}$. It is worth noting that the correlation between threshold in fidelity and $t_{CNOT}$ is not clear, so relaxation of the threshold would not necessarily lead to the faster CNOT operation.

**Figure 5:**
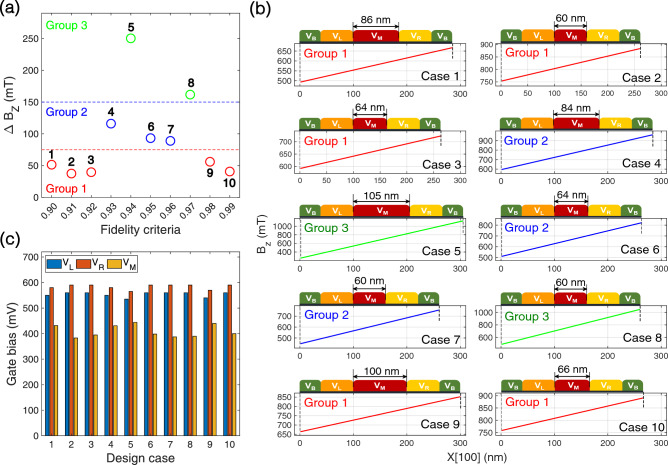
Physical design of double quantum dot (DQD) structures that reproduce solutions driven by the Bayesian optimization (BO) process. (**a**) Difference of magnitudes in magnetic field ($\Delta B_Z$) at the left and right QD spot. The two magnitudes are obtained by converting Zeeman-splitting energies ($E_{ZR}$ and $E_{ZL}$). $\Delta B_Z$’s are plotted with circles that are numbered based on the fidelity criteria such that ‘1’, ‘2’, ..., ‘10’ indicate the case of 90%, 91%, ..., 99%, respectively. Design cases are categorized into three groups, where $B_Z$ profiles in each group have the same gradient along the lateral (*X*) direction. (**b**) $B_Z$ profiles along the *X* ([100]) direction and corresponding sizes of the middle gate ($L_M$) in 10 design cases. Gate electrodes are also schematically illustrated to show that we only changed $L_M$ to drive $B_Z$ profiles that reproduce BO-driven $E_{ZR}$ and $E_{ZL}$, using the structure shown in Fig. 3a as a baseline. (**c**) DC biases of left ($V_L$), middle ($V_M$), and right gate ($V_R$) that symmetrically initialize the DQD system to a $|\downarrow \downarrow \rangle$ state and reproduce BO-driven exchange energies (*J*).

So far we have checked that BO delivers optimal values of $E_{ZL}$, $E_{ZR}$ and *J* satisfying the criteria of *F*. However, BO does not provide any clues on whether the optimal solutions are realizable with physical devices, so another essential task of this work should be to verify the practicality of our design framework by securing physical designs of Si DQD devices that produce the optimal solutions obtained with BO. The first step for device designs here would be to decide the distribution of a static magnetic field ($B_Z$), which are externally applied to the DQD system in reality^25^. Assuming that $B_Z$ changes linearly along the lateral ([100], *X*) direction (see Fig. 3a), we try to determine the distance between two QDs such that $E_{ZL}$ and $E_{ZR}$ obtained with BO can be reproduced reasonably. For this purpose, we convert $E_{ZL}$ & $E_{ZR}$ to the magnitude of $B_Z$ using the Bohr magneton constant for electron spin momentum ($\sim$5.78$\times$10^-5^ eV/T), and plot the difference of two $B_Z$’s ($\Delta B_Z$) in Fig. 5a, where circles are numbered from 1 to 10 in accordance with fidelity criteria in an ascending order, *i*.*e*., ‘1’, ‘2’, ..., and ‘10’ correspond to the design case of *F* $\ge$ 90%, 91%, ..., and 99%, respectively. Since $\Delta B_Z$ ranges from about 35mT to 250mT, the lateral distance between two QDs in design cases can vary up to 7 times with a single gradient ($\partial B_Z/\partial X$), and it may be hard to secure *J* strong enough to implement fast CNOT operations if QDs are too far. Accordingly, we categorize the design cases into 3 groups based on the magnitude of $\Delta B_Z$, employing different gradients (0.48 mT/nm, 1.15 mT/nm, and 2.10 mT/nm for group 1, 2, and 3, respectively). For structural designs of the DQD system, we only vary the size of the middle gate ($L_M$) from the original DQD structure shown in Fig. 3a, setting $L_M$ to 60 nm in the baseline case of each group that has the smallest $\Delta B_Z$. Figure 5b shows the results with lateral distributions of $B_Z$ and the length of middle gates to scale in all the design cases. Corresponding sets of ($V_L$, $V_R$, $V_M$), which initialize the system with symmetric biasing, are presented in Fig. 5c ($V_B$ is set to 200 mV for all the cases).

**Figure 6:**
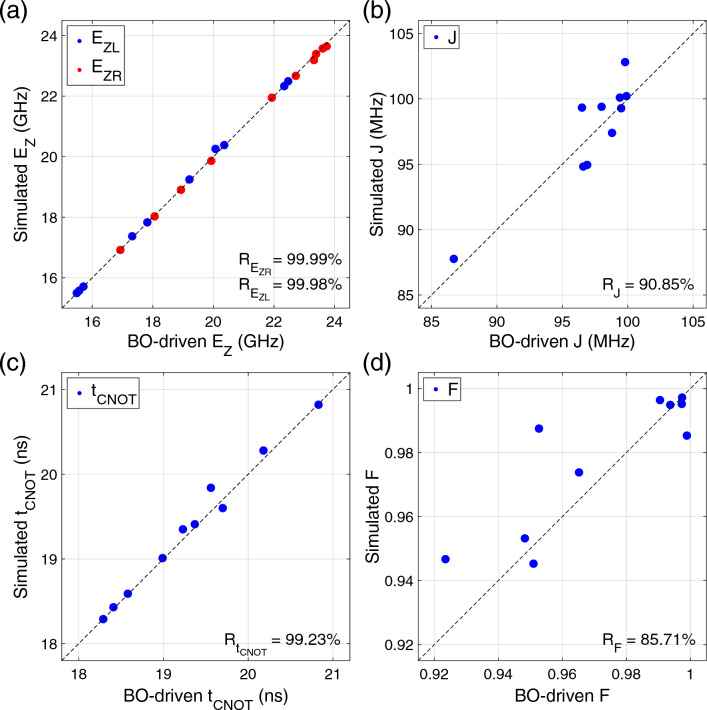
Correlation between simulated and BO-driven results. Simulated results obtained with secured physical designs are compared with solutions obtained with BO; Zeeman-splitting energy ($E_{ZL}$, $E_{ZR}$) in (**a**), exchange energy (*J*) in (**b**), CNOT operation time ($t_{CNOT}$) in (**c**), and gate fidelity (*F*) in (**d**). An outstanding linear correlation is observed between simulated results and BO solutions in cases of $E_{ZL}$, $E_{ZR}$ and $t_{CNOT}$, while a slightly weaker correlation is found in cases of *J* and *F*.

To examine if the BO-driven results ($E_{ZL}$, $E_{ZR}$, *J*, $t_{CNOT}$, *F*) can be secured in physical systems, we conduct device simulations against Si DQD structures (Fig. 5b) with bias points (Fig. 5c), and show the correlation between simulated and BO-driven results in Fig. 6a and b. The correlation coefficient (*R*) turns out to be quite strong ($\simeq$ 99.98% and $\simeq$ 99.94%) in the case of $E_{ZL}$ and $E_{ZR}$. *R* of *J* ($\simeq$ 90.85%) is also nice, but is a bit weaker than in the case of $E_{ZL}$ and $E_{ZR}$. The reason why *J* has weaker correlation can be explained with the huge sensitivity that *J* has against $V_M$. Since *J* fluctuates by a factor of up to several hundreds with even a few mV of $\Delta V_M$ as we reported^25^, very tiny mismatches in $L_M$ and $V_M$ can drive significant deviations of *J*, and it is therefore generally challenging to make the correlation of *J* as strong as that of $E_{ZL}$ and $E_{ZR}$. Figure 6c and d show speed and fidelity of CNOT operations, respectively. Results here generally turn out to be solid enough to claim the strong correlation in the case of $t_{CNOT}$ (*R* $\simeq$ 99.23%) and *F* (*R* $\simeq$ 85.71%). Again, the origin of the relatively worse correlation that *F* shows, can be found from the fact that the fidelity of a single-step CNOT operation implemented in the Si DQD platform strongly depends on *J* (and so $V_M$)^25,39^. Despite some fluctuations in correlation between simulated and BO-driven results, derived *R*’s can generally support the practicality of our design framework, which is still valid for results secured with a threshold of 99.9% and 99.99% (see the Supplementary Information document).

## Conclusion

An engineering framework based on the Bayesian optimization (BO) is proposed for implementation of quantum logic operations in electrode-driven quantum dot (QD) systems in silicon (Si). With our in-house device simulation code that solves electrostatics of Si QD structures and corresponding time responses of quantum bits (qubits) encoded to electron spins, we get the initial design of a Si double QD (DQD) structure that gives Zeeman-splitting energies ($E_Z$) and exchange energy (*J*) with which the experimentally reported controlled-X (CNOT) operation^12^ is well mimicked. BO is then conducted to find unknown sets of ($E_Z$, *J*) that maximize the speed of CNOT operations whilst maintaining the operational fidelity larger than user-defined criteria ranging from 90% to 99%. The optimal solutions procured by the BO process turn out to improve the performance of 2-qubit CNOT logic implemented in the Si DQD system such that all the searched solutions complete the operation within around 20 nsec with the fidelity $\ge$ 90%, which is almost 5 times faster than the initial design that mimics the experimental result. The BO-driven solutions are validated by finding physical designs of Si DQD structure, for which we elaborately adjusted sizes & biases of gate electrodes together with distribution of static magnetic field such that ($E_Z$, *J*) obtained with BO can be faithfully reproduced with device simulations. For all the design cases we considered, the correlation between BO-driven solutions and results of device simulations turns out to be quite strong. Devising a systematic design framework that combines BO with our in-house multi-scale modeling approach of semiconductor devices^25,28^, this work contributes to opening a practical way for optimal designs of complicated multi-qubit operations with electrically defined semiconductor QD platforms.

### Supplementary Information

Supplementary Information.

## Data Availability

The datasets generated and analysed during the current study are available from the corresponding author on reasonable request.
