# Supplementary material for: Design of high-performance entangling logic in silicon quantum dot systems with Bayesian optimization
Source: Sci Rep. 2024 May 2;14:10080. doi: 10.1038/s41598-024-60478-9 (PMC11066012; doi:10.1038/s41598-024-60478-9)
Supplement: Supplementary file 1 — Supplementary Information. [file 41598_2024_60478_MOESM1_ESM.pdf]

Design of high-performance entangling logic in silicon quantum dot systems with Bayesian optimization

- Supplementary Information -

Ji-Hoon Kang [1,‡], Taehyun Yoon [2,‡], Chanhui Lee [3], Sungbin Lim [4,*], and Hoon Ryu [1,*]

[1] Div. of National Supercomputing, Korea Institute of Science and Technology Information, Daejeon 34141, Republic of Korea.

[2] Artificial Intelligence Graduate School, Ulsan National Institute of Science and Technology, Ulsan 44919, Republic of Korea

[3] Department of Artificial Intelligence, Korea University, Seoul 02841, Republic of Korea.

[4] Department of Statistics, Korea University, Seoul 02841, Republic of Korea.

[‡] These authors equally contributed to this work.

[*] Corresponding authors: sungbin@korea.ac.kr (S. Lim), elec1020@kisti.re.kr (H. Ryu)

1. Enhancing fidelity conditions

Further searches on design solutions that satisfies the fortified fidelity (F) conditions, $F \geq 99.9\%$ and $F \geq 99.99\%$, are conducted to verify the effectiveness of the proposed design framework in the regime of more challenging conditions. Using the Bayesian optimization (BO) in the present approach, the optimal design parameters are obtained as (E_{ZL}, E_{ZR}, J) = (22.164 GHz, 22.983 GHz, 73.257 MHz) for $F \geq 99.9\%$ and (E_{ZL}, E_{ZR}, J) = (16.494 GHz, 23.928 GHz, 54.197 MHz) for $F \geq 99.99\%$.

As we did in the main manuscript, here we conduct device simulations to check whether it is possible to secure physical designs of Si double quantum dot systems that produce the BO-driven parameters presented in the previous paragraph. Figure S1(a) shows the device structures that are simulated to reproduce design parameters with a fidelity threshold of 99.9% (left) and 99.99% (right). Note that the BO- & device-driven design parameters with corresponding t_{CNOT} and F, which are summarized in Figure S1(b), turn out to be close enough to clearly support the practicality of our proposed framework for quantum logic device designs.

(a)

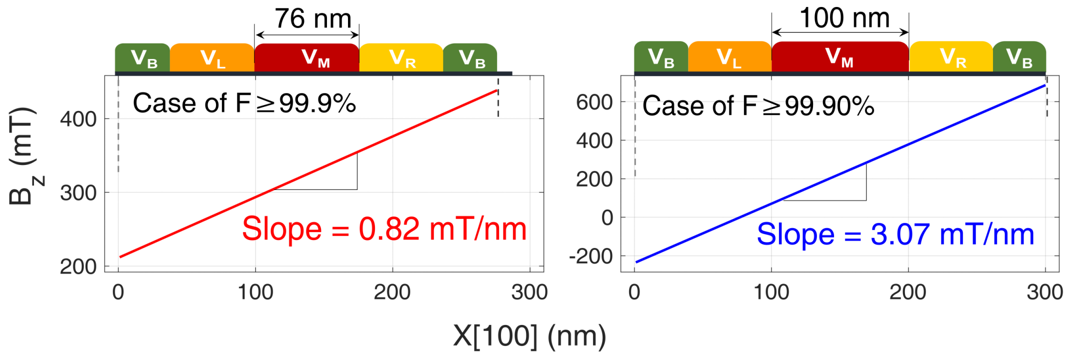

(b)

Design condition		E_{ZL} (GHz)	E_{ZR} (GHz)	J (MHz)	t_{CNOT} (nsec)	F (%)
$F \geq 99.9\%$	BO driven	22.164	23.983	73.257	26.333	99.941
	Simulated	22.164	23.997	73.162	26.363	99.927
	Difference	0%	0.058%	0.130%	0.114%	0.014%
$F \geq 99.99\%$	BO driven	16.494	23.928	54.197	35.727	99.999
	Simulated	16.483	23.994	55.495	35.751	99.937
	Difference	0.067%	0.276%	2.395%	0.663%	0.062%

Figure S1. (a) The Si double quantum dot structure that is considered to reproduce BO-driven parameters when the fidelity threshold is set to for $F \geq 99.9\%$ (left) and $F \geq 99.99\%$ (right). **(b)** The BO- & simulation-driven design parameters (E_{ZL}, E_{ZR}, J) and their output quantities (t_{CNOT} and F). Note that device-driven results are solid enough to claim the practicality of our proposed design approach. The employed bias conditions are (V_L, V_M, V_R) = (555mV, 419.7mV, 585mV) for $F \geq 99.9\%$ and (V_L, V_M, V_R) = (525mV, 433.9mV, 555mV) mV for $F \geq 99.99\%$, respectively.
